# Detecting Left Heart Failure in Echocardiography through Machine Learning: A Systematic Review

**DOI:** 10.31083/j.rcm2312402

**Published:** 2022-12-12

**Authors:** Lies Dina Liastuti, Bambang Budi Siswanto, Renan Sukmawan, Wisnu Jatmiko, Yosilia Nursakina, Rindayu Yusticia Indira Putri, Grafika Jati, Aqsha Azhary Nur

**Affiliations:** ^1^Department of Cardiology and Vascular Medicine, Faculty of Medicine Universitas Indonesia, National Cardiovascular Center Harapan Kita Hospital, 15810 Jakarta, Indonesia; ^2^Department of Cardiology and Vascular Medicine, Faculty of Medicine Universitas Indonesia, Dr. Cipto Mangunkusumo Hospital, 10430 Jakarta, Indonesia; ^3^Department of Computer Science, Faculty of Computer Science Universitas Indonesia, 16424 Depok, Indonesia; ^4^School of Public Health, Imperial College London, SW7 2BX London, UK; ^5^The Johns Hopkins Bloomberg School of Public Health, Baltimore, MD 21205, USA

**Keywords:** heart failure, echocardiography, machine learning

## Abstract

**Background:**

Heart failure remains a considerable burden to healthcare in Asia. Early intervention, mainly using echocardiography, to assess cardiac function is crucial. However, due to limited resources and time, the procedure has become more challenging during the COVID-19 pandemic. On the other hand, studies have shown that artificial intelligence (AI) is highly potential in complementing the work of clinicians to diagnose heart failure accurately and rapidly.

**Methods:**

We systematically searched Europe PMC, ProQuest, Science Direct, PubMed, and IEEE following the Preferred Reporting Items for Systematic Reviews and Meta-Analyses (PRISMA) guidelines and our inclusion and exclusion criteria. The 14 selected works of literature were then assessed for their quality and risk of bias using the QUADAS-2 (Quality Assessment of Diagnostic Accuracy Studies).

**Results:**

A total of 2105 studies were retrieved, and 14 were included in the analysis. Five studies posed risks of bias. Nearly all studies included datasets in the form of 3D (three dimensional) or 2D (two dimensional) images, along with apical four-chamber (A4C) and apical two-chamber (A2C) being the most common echocardiography views used. The machine learning algorithm for each study differs, with the convolutional neural network as the most common method used. The accuracy varies from 57% to 99.3%.

**Conclusions:**

To conclude, current evidence suggests that the application of AI leads to a better and faster diagnosis of left heart failure through echocardiography. However, the presence of clinicians is still irreplaceable during diagnostic processes and overall clinical care; thus, AI only serves as complementary assistance for clinicians.

## 1. Introduction

Heart failure (HF) remains a significant global health problem leading to high hospitalization and mortality rate despite advances in therapy [1]. The burden of the disease in Asia is particularly more pronounced, considering that it affects a younger population than in Europe and America [2, 3]. Early detection and treatment of possible cases are mandatory to prevent disease progression and reduce health care costs.

Echocardiography is a widely recommended imaging modality for assessing cardiac function in HF patients [4, 5]. Although echocardiography is non-invasive, harmless, and relatively inexpensive, some severe issues have arisen regarding its implementation. Echocardiography test is largely dependent on the user’s skill, creating challenges for interpretation [6]. Furthermore, the terminology of left HF comprises a wide range of phenotypes, from those with systolic dysfunction or reduced ejection fraction (HFrEF) [EF <40%], diastolic dysfunction or preserved ejection fraction (HFpEF) [EF ≥50%], and the ‘grey area’ cases with mid-range ejection fraction (HFmrEF) [EF 40–49%] [5]. Diagnosing HFpEF from echocardiography alone is not a simple task as the European Society of Cardiology guidelines recommends combining with other diagnostic tests, including natriuretic peptides level and electrocardiogram (ECG) [5, 7].

The most potential solution to the limitation of echocardiography interpretation lies in the application of automated methods, which have vastly evolved through computer technology. Artificial intelligence leverages computers and machines to mimic the human mind in problem-solving capacities. It enables training of large databases of various echocardiographic videos and images which have been previously confirmed by experts to achieve knowledge which is then used to identify endocardial pathologies in other cases [8].

### The Role of Machine Learning

Machine learning (ML)—a domain of artificial intelligence (AI) described as a computer for learning from experiences to perform prearranged tasks without preceding knowledge—has recently been used to improve diagnostic analysis in the medical field, notably in imaging modalities [9]. The development of ML has made a considerable leap to help with multiple tasks, including pattern identification, classification, and calculation [10].

There are two main types of the algorithm within the field of ML: supervised and unsupervised ML. Supervised ML aims to train models capable of predicting the output of labeled data, whereas unsupervised ML refers to analyses that learn from unlabelled data to find hidden patterns and practical insights. Supervised ML encompasses classification and regression functions. In echocardiography, the classification function is beneficial for determining the presence or absence of a disease, while the regression function is widely used to calculate exact values, such as left atrial pressure. Unsupervised ML application in echocardiography is mainly implemented in clustering and dimensionality reduction problems. The clustering algorithm operates by grouping cases based on their similarity. The dimensionality reduction ameliorates data complexity, thereby increasing visualization and interpretability, creating a better dataset version for subsequent ML processes. In some cases, the subtypes of ML can be combined to produce an even more robust algorithm, such as deep reinforcement learning [9, 11].

Studies related to ML for diagnosing systolic and diastolic dysfunction have proliferated. Various algorithms were trained and tested, resulting in diverse diagnostic accuracy. Nevertheless, no study systematically reviews the available works of literature on this issue. Thus, in this systematic review, we aim to investigate the best practice of ML for echocardiography dataset analysis in the diagnosis of heart failure.

## 2. Materials and Methods

### 2.1 Search Strategy, Selection Criteria, and Study Selection

We systematically searched Europe PMC, ProQuest, Science Direct, PubMed, and IEEE with the search terms (“artificial intelligence” OR “machine learning” OR “deep learning”) AND “echocardiography” AND (“ejection fraction” OR “left heart failure” OR “systolic” OR “diastolic”) AND (“sensitivity” OR “specificity” OR “accuracy”). Other pieces of literature are found through hand searching. The study was conducted following the Preferred Reporting Items for Systematic Reviews and Meta-Analyses (PRISMA) guidelines. We used the following inclusion criteria: (1) original studies (e.g., cohort, cross-sectional, diagnostic study) conducted in normal and heart failure patients, (2) available in full text and English language, (3) published in the last ten years, (4) has outcomes of accuracy measures, e.g., sensitivity, specificity, and area under the curve (AUC), (5) contains echocardiography video/image data as their training and testing dataset, and (6) has specified the ML technique used in the study.

We excluded articles that do not match our PICO (Population, Intervention, Comparison, Outcome) and those which were non-original research, not available in full-text, non-English language articles, and those that integrate echocardiography with other parameters. The incorporation of other clinical parameters into the machine would introduce bias and hinder the machine to learn to distinguish HF and non-HF patients based on echocardiographic images alone. Based on the above inclusion and exclusion criteria, two reviewers independently screened article titles and abstracts of the identified eligible articles. Full-text screening, retrieved through institutional access, was done to ensure the relevance of the articles. Experts resolved any discrepancies during this process in the related field. The search was finalized on February 25th, 2022.

### 2.2 Data Extraction, Data Synthesis, and Quality Assessment

Data extraction was done independently by two reviewers after verification by two senior authors. We extracted each study’s data items in a tabulated format: author (year), study objective, population, data type, echocardiography view, machine learning algorithm, machine learning scenario, and results. The quality and risk of bias of the included studies were assessed using the QUADAS-2 (Quality Assessment of Diagnostic Accuracy Studies), an assessment tool to determine the quality of diagnostic accuracy study. It includes the risk of bias and applicability concerns in patient selection, index test, reference standard, flow, and timing. The QUADAS-2 tool is implemented in 4 phases: summarizing the review questions, adapting the tool and generating review-specific guidelines, compiling a flowchart for the main study, and assessing bias and applicability [12].

## 3. Results

**Fig. 1.**
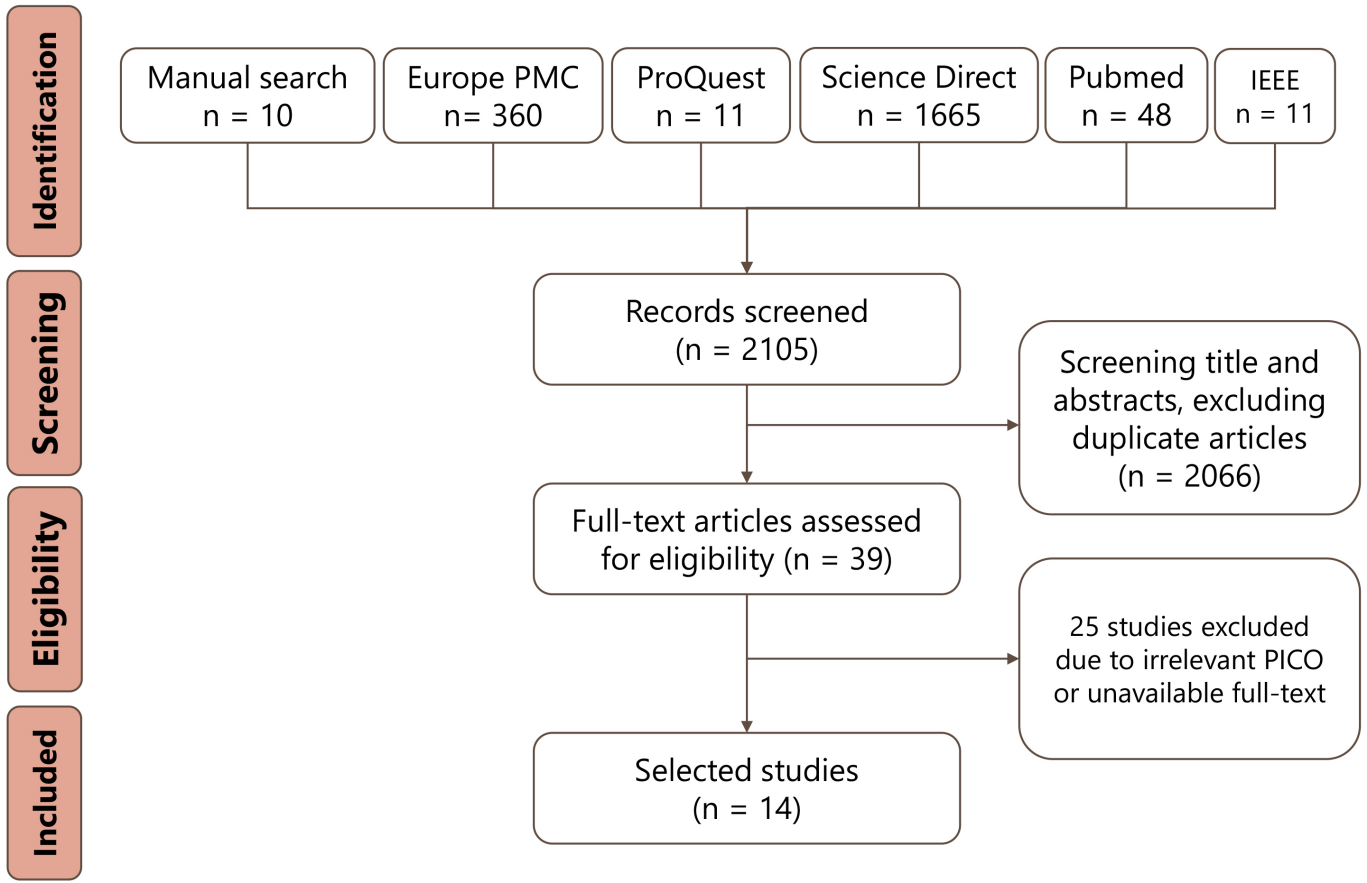
**PRISMA flow diagram of study selection process**.

A total of 2105 citations were retrieved by the method aforementioned. After reading titles and abstracts and assessing these articles for eligibility, 2066 citations were excluded. Full-text articles were assessed, and 25 articles were excluded as these literatures did not match our PICO or were duplicate articles. As a result, 14 articles remained and included in this systematic review [6, 13, 14, 15, 16, 17, 18, 19, 20, 21, 22, 23, 24, 25, 26, 27]. The detailed elaboration of PRISMA flow is described in Fig. 1.

Only a few studies pose unclear risk of bias in terms of patient selection, including Dong *et al*., Ghorbani *et al*., Behnami *et al*., and Liu *et al*. These risk of bias was caused by unclear inclusion and exclusion criteria and an unspecified randomization process. Studies with unclear risk of bias in their index test, including Dong *et al*., Ghorbani *et al*., Bhenami *et al*., Chiou *et al*., Kusunose *et al*., Liu *et al*., and Chen *et al*., demonstrated ambiguity of blinding process. Other studies exhibit low risk of bias and low applicability concern in all of the QUADAS-2 variables. Complete QUADAS-2 assessment of these literatures are explained in Table 1 [6, 13, 14, 15, 16, 17, 19, 20, 22, 23, 24, 25, 26, 27].

**Table 1.** **Quality assessment of the included literatures**.

No	Author	Risk of bias				Applicability Concerns		
		Patient selection	Index test	Reference standard	Flow and timing	Patient selection	Index test	Reference standard
1	Dong, *et al*. (2016) [13]	Unclear	Unclear	Low	Low	Low	Low	Low
2	Raghavendra, *et al*. (2017) [14]	Low	Low	Low	Low	Low	Low	Low
3	Sanchez-Martinez, *et al*. (2018) [15]	Low	Low	Low	Low	Low	Low	Low
4	Tabassian, *et al*. (2018) [17]	Low	Low	Low	Low	Low	Low	Low
5	Silva, *et al*. (2018) [16]	Low	Low	Low	Low	Low	Low	Low
6	Ouyang, *et al. *(2020) [19]	Low	Low	Low	Low	Low	Low	Low
7	Ghorbani, *et al*. (2020) [20]	Unclear	Unclear	Low	Low	Low	Low	Low
8	Behnami, *et al*. (2019) [22]	Unclear	Unclear	Low	Low	Low	Low	Low
9	Chiou, *et al*. (2021) [23]	Low	Unclear	Low	Low	Low	Low	Low
10	Kusunose, *et al*. (2021) [24]	Low	Unclear	Low	Low	Low	Low	Low
11	Liu, *et al*. (2021) [25]	Unclear	Unclear	Low	Low	Low	Low	Low
12	Pandey, *et al*. (2021) [26]	Low	Low	Low	Low	Low	Low	Low
13	Chen, *et al. *(2021) [27]	Low	Unclear	Low	Low	Low	Low	Low
14	Tromp, *et al*. (2022) [6]	Low	Low	Low	Low	Low	Low	Low

The characteristics and results of these 14 studies are summarised in Table 2 [6, 13, 14, 15, 16, 17, 19, 20, 22, 23, 24, 25, 26, 27]. Nearly all datasets are in 3D or 2D images, and the most common echocardiography views used in training are apical four-chamber (A4C) and apical two-chamber (A2C). Supervised ML is frequently used as the algorithm’s core, notably convolutional neural network, to classify and differentiate individuals with and without the disease.

**Table 2.** **Machine learning utilization in evaluation of left heart failure**.

Author (year)	Objectives	Population	Data type	View/variables	Machine learning task/algorithm	Result
Dong, *et al*. (2016) [13]	Automated LVEF measurement compared to manual measurement	**Internal dataset**	3D image	Not reported	**Feature extraction**	Good correlation between both methods for LVEDV (r = 0.85), LVESV (r = 0.871), & LVEF (r = 0.863)
		60 cases for unsupervised ML & 120 cases for supervised ML			Multi-scale convolutional deep network (unsupervised)	
		**External dataset**			**Volume estimation**	
		120 cases of various LVEF			Random forest (supervised)	
Raghavendra, *et al*. (2017) [14]	CHF diagnosis determination compared to clinical judgement	50 CHF cases & 50 normal samples	2D image	A4C	**Feature extraction**	High diagnostic value, including 99.33% accuracy, 98.66% sensitivity, 100% specificity and 100% PPV
					Variational Mode Decomposition	
					**Feature selection**	
					Particle Swarm Optimization	
					**Classification**	
					SVM	
Sanchez-Martinez, *et al*. (2018) [15]	HFpEF diagnosis determination compared to clinical judgement	**Internal dataset**	2D image & Doppler modalities	A4C /total 24 variables	**Reduction**	**Internal dataset**
		72 HFpEF cases & 33 healthy controls			Multiple kernel learning (unsupervised)	Moderate agreement between ML and clinical diagnosis (κ, 72.6%; 95% CI, 58.1–87.0)
		**External dataset**			**Clustering**	**External dataset**
		27 breathless & 24 hypertensive patients			Agglomerative hierarchical clustering (unsupervised)	Misclassification of 33% hypertensive & 67% breathless cases as mild-HFpEF
Tabassian, *et al*. (2018) [17]	HFpEF diagnosis determination compared to clinical judgement	33 HFpEF cases, 67 controls (healthy, breathless, hypertensive group) during rest & exercise	Doppler modalities	Velocity (4 variables), strain (3 variables), & strain rate (5 variables)	**Missing data imputation**	- The overall accuracy of patient classification was the highest using strain rate (57%), compared to velocity (50%) and strain data (31%)
					KNNimpute (supervised)	
					**Pattern learning**	
					PCA (unsupervised)	- The highest HFpEF diagnostic accuracy (81%) was achieved by strain rate measurement
					**Classification**	
					DWKNN (supervised)	
Silva, *et al*. (2018) [16]	Automated EF classification compared to manual measurement	**Internal dataset**	2D image of TEE	A4C	3D-CNN (supervised)	- High accuracy of 78%
		4000 cases for training				- F1 score of 71.3% for unhealthy EF (below 45%), 63.3% for intermediate EF (45–55%), 72.3% for healthy EF (55–75%) and 54.6% for abnormally high EF (above 75%)
		**External dataset**				
		1600 cases for testing				
Ouyang, *et al*. (2020) [19]	Cardiac segmentation & LVEF measurement accuracy	**Internal dataset**	2D image	A4C	EchoNet (CNN/supervised)	**Internal dataset**
		10,030 echo-indicated cases				- High correlation in LV segmentation of ESV (Dice coefficient 0.903) & EDV (Dice coefficient 0.927)
						- High diagnostic value (AUC 0.97) to predict LVEF <50% (cardiomyopathy)
		**External dataset**				**External dataset**
		2895 echo-indicated cases				High diagnostic value (AUC 0.96) to predict LVEF <50% (cardiomyopathy)
Ghorbani, *et al*. (2020) [20]	Diagnosing anatomic features and cardiac function	3312 cases of wide variety of cardiac diseases	2D image	A4C	EchoNet (CNN/supervised)	- High accuracy in predicting anatomical condition, including pacemaker ((AUC of 0.89, F1 score of 0.73), severely dilated left atrium (AUC of 0.85, F1 score of 0.68), and left ventricular hypertrophy (AUC of 0.75, F1 score of 0.57)
						- Good accuracy in predicting LVESV ($R^{2}$ = 0.74), LVEDV ($R^{2}$ = 0.7), and LVEF ($R^{2}$ = 0.5)
Behnami, *et al*. (2020) [22]	Automated EF classification compared to manual measurement	645 cases with moderate EF & 541 cases with reduced EF	2D image	A2C & A4C	**Feature extraction**	High overall accuracy of 83.15%, precision of 82.6% and recall of 81.1%
					DenseNet (CNN/supervised)	
					**Sequence processing**	
					bi-GRU (RNN/supervised)	
Chiou, *et al*. (2021) [23]	HFpEF diagnosis determination compared to clinical judgement	**Internal dataset**	2D image	A4C	**Image segmentation**	**Internal dataset**
		1041 HFpEF cases & 1263 controls			U-Net (supervised)	LA area calculation showed high accuracy (91%), sensitivity (96%), specificity (85%), and AUC (0.95) for HFpEF classification
		**External dataset**			**Classification**	**External dataset**
		150 COPD & 315 HFpEF cases			1D CNN (supervised)	LV area calculation showed high accuracy (87%), sensitivity (84%), specificity (88%), and AUC (0.93) for HFpEF classification
Kusunose, *et al*. (2021) [24]	Echo-view classification & accuracy of EF measurement	**Internal dataset**	2D image	A2C, A3C, A4C, PLAX, PSAX	**View classification**	**External dataset**
		340 patients with various LVEF			CNN (supervised)	- 98.1% accuracy for echo-view classification
		**External dataset**			**EF Classification**	- Good correlation between reference & estimated EF (r = 0.8 to 0.82)
		189 patients			3D-CNN (supervised)	
Liu, *et al*. (2021) [25]	Echo-view classification, accuracy & consistency of EF measurement	**Version 1 (Internal dataset)**	2D image	**Version 1**	**DPS-Net v1**	**Version 1**
		100 echo-indicated cases		A2C, A3C, A4C	CNN based on a modified U-net (supervised)	- Good performance of echo view classification (Dice coefficient A2C = 0.931; A3C = 0.931; A4C = 0.933)
		**Version 1 (external data set)**				
		240 echo indicated-cases				
						- High accuracy (90%), sensitivity (91%), specificity (89%), PPV (86%), NPV (93%) of LVEF measurement
						- High consistency (ICC = 0.998, MAE 1.2%) of LVEF measurement
		**Version 2**		**Version 2**	**DPS-Net v2**	**Version 2**
		10,530 echo-indicated cases		Additional A2C, A4C	CNN based on a modified U-net (supervised)	- Better performance of LV segmentation and echo-view classification (Dice coefficient = 0.935)
						- Good agreement of LVEF measurement with means ± SD of 1.70% ± 4.13% with 95% CI (9.79, 6.39).
Pandey, *et al*. (2021) [26]	Detection of HFpEF compared to 2016 ASE guideline (gold standard: elevated LV filling pressure during cardiac catheterization)	**Internal dataset**	2D image & Doppler modalities	EF, LV mass index, E, A, E/A ratio, e’, E/e’ ratio, LA volume index, tricuspid regurgitation peak	**Clustering**	**Internal dataset**
		1242 cases with various systolic & diastolic function			TDA network (unsupervised)	High diagnostic accuracy in predicting low risk and high risk group (AUC 0.997. accuracy 96%)
		**External dataset**			**Classification**	**External dataset**
		83 cases for detection of elevated LV filling pressure			DeepNN (supervised)	- High risk group correlated with higher LV filling pressure (r = 0.76; *p *< 0.0001)
						- Higher diagnostic value than 2016 guideline for predicting elevated LV filling pressure (AUC 0.88 vs 0.67)
Chen, *et al*. (2021) [27]	ALHF diagnosis determination compared to clinical judgement (echo & ML vs echo only)	80 cases of various grade of ALHF	2D image	Not reported	Deep CNN (supervised)	Diagnostic coincidence rate of patients from the echo & ML vs echo only group was 93.94% vs 74.29%
			Doppler modalities	E, A, E/A		
Tromp, *et al*. (2022) [6]	Echo-view classification & LV systolic - diastolic function measurement	**Internal dataset**	2D video	A2C, A4C, PLAX	**View classification**	**Internal dataset**
		1145 cases	Doppler modalities	PWTDI, M-mode, pulse wave, continuous wave	CNN (supervised) & auto encoder (unsupervised)	- High accuracy of echo-view classification, ranging from 91.1% for PWTDI to 98.9% for PLAX.
						- High correlation of the measurement of LVEF (r = 0.89, MAE 5.5%)
						- High diagnostic value of identifying systolic dysfunction (LVEF < 40%, AUC 0.96) & diastolic dysfunction (E/e’ ratio ≥ 13, AUC 0.96)
		**External dataset**				**External dataset**
		3 multicentre data of total 42,300 cases				High diagnostic value for identifying systolic dysfunction (AUC range 0.90-0.92) & diastolic dysfunction (AUC range 0.91-0.91)

ASE, American Society of Echocardiography; ALHF, acute left heart failure; CHF, congestive heart failure; HFpEF, heart failure with preserved ejection fraction; 2D, 2-dimensional; A4C, apical four chamber; A3C, apical three chamber; A2C, apical two chamber; PLAX, parasternal long axis; PSAX, parasternal short axis; PWTDI, pulse wave tissue Doppler imaging; LA, left atrium; LV, left ventricle; RV, right ventricle; EF, ejection fraction; ES, end systolic; ED, end diastolic; LVESV, left ventricle end systolic volume; LVEDV, left ventricle end diastolic volume; LAESV, left atrium end systolic volume; E, early diastolic transmitral flow velocity; A, late diastolic transmitral flow velocity; e’, early diastolic relaxation velocity; CNN, convolutional neural networks; RNN, recurrent neural networks; SVM, support vector machine; PCA, principal-component analysis; DWKNN, distance-weighted k-nearest-neighbor; STRE, spatiotemporal-rest-exercise; TDA, topological data analysis; PPV, positive predictive value; NPV, negative predictive value; MAE, mean absolute error; ICC, interclass correlation coefficient; TTE, transthoracic echocardiograms.

Dong *et al*. [13] proposed a method incorporating unsupervised multi-scale convolutional deep networks and random forests to predict LV volume and calculate LVEF. The multi-scale convolution deep network extracted features of unlabelled end-diastolic and end-systolic 3DE volumes (EDV and ESV). Afterward, the left ventricular volume was formulated as a regression problem; thus, random forests were used to estimate the efficient volume.

Raghavendra *et al*. [14] developed a framework of ML techniques to classify CHF due to dilated cardiomyopathy and normal controls. The 2-D images were decomposed to generate specific structural patterns of each group using variational image decomposition (VMD). After the texture feature is extracted and enhanced using particle swarm optimization (PSO), the support vector machine (SVM) separates the class members into two groups.

Sanchez-Martinez *et al*. [15] combined unsupervised ML algorithms to investigate left ventricular long-axis myocardial velocity patterns that ordered subjects according to their similarities, allowing further analysis of the main trends in velocity patterns. The clustering system identified a continuum from normal to HF, including a transition zone of uncertain diagnosis. This method was subsequently independently validated in two additional cohorts, breathless and hypertensive patients. These resulted in limited accuracy and misclassification into the HFpEF group.

Almost all recent studies adopted CNN (convolutional neural networks) as the principal classifier, but each has modifications to elevate diagnostic power. Silva* et al*. [16] demonstrated custom 3D-CNN ability to integrate temporal knowledge from transthoracic echocardiography (TTE) cine loops to calculate LVEF and classified it into four classes. Ouyang *et al*. [19] and Ghorbani *et al*. [20] presented a novel CNN-based ML called Echo-Net. The algorithm performed several tasks, from left ventricle segmentation during systole and diastole, beat-to-beat prediction of the ejection fraction, and presence of heart failure conclusion. The ML was also able to identify the local cardiac structures and anomalies, measure volumetric parameters and metrics of cardiac function, and predict systemic human phenotypes that modify cardiovascular risk. Behnami *et al*. [22] built combined supervised ML for binary EF classification without segmentation. What is even more interesting is that two image views, A4C and A2C, were concatenated for temporal embedding. Kusunose *et al*. [24] compared two types of input method averaged images and ten selected images from 5 standard views and tested them using 3D-CNN to recognize the view type and estimate LVEF. The group with selected images improved the overall accuracy of echo-view classification and LVEF estimation.

Several studies carried out diagnostic classification after image segmentation using U-net. Chiou *et al*. [23] used U-net for left atrium and left ventricle segmentation to measure their length, width, area, and volume. The interbeat dynamic changes were then recorded as linear waveform signals, trained and classified by a 1D CNN. Liu *et al*. [25] presented a DPS-Net model, a constructed CNN based on modified U-net, and tested the ML on a local dataset of A2C, A3C, and A4C images. After that, the algorithm was retrained using a sizeable multicenter dataset to generate better accuracy in view classification, end-systolic and end-diastolic frame detection, and by all means, LVEF measurement.

ML analyses using datasets of the Doppler modality have recently become more promising. Tabassian *et al*. [17] investigated spatiotemporal characteristics of velocity, strain, and strain rate traces during rest and exercise from tissue Doppler using a supervised and unsupervised algorithm. Each parameter of the rest and exercise tests was concatenated, and the pattern was analyzed using principal component analysis (PCA). Further, automatic classification using distance-weighted ĸ-nearest-neighbor (DWKNN) was applied to differentiate HFpEF cases and multi-phenotype controls. Pandey *et al*. [26] developed a combination of unsupervised and supervised learning and trained the algorithm using the dataset of routinely measured Doppler indexes. The model was also implemented in the hemodynamic external validation cohort to identify two phenogroups (high-risk vs. low-risk) patients and demonstrated a strong diagnostic value. Chen* et al*. [27] tested the performance of Deep CNN, which was predicted to have better application recognition performance due to more layers in the ML architecture and simplified connection. Aside from 2D images data, Doppler indexes, including atrial systolic velocity (A), early mitral valve diastolic maximum velocity (E), were also processed to enhance the diagnostic accuracy. The latest study is from Tromp *et al*. [6], which presented a new ML approach of 2D videos and Doppler parameters that allows fully automated classification and annotation of echocardiographic videos. These 2D videos were classified into views by two different classifiers, a supervised CNN or an unsupervised deep clustering CNN. Meanwhile, the Doppler modalities view classifier consisted of integrated CNN models trained with the echo or velocity trace images and the categorical ground truth labels.

## 4. Discussion

Our systematic review represents the model’s current state for diagnosing heart failure more rapidly through echocardiography images. The literature included in this study has shown that AI has comparable performance in characterizing heart failure through echocardiography images, compared with the conventional method by medical practitioners, with an accuracy rate ranging from 57% to 99.3%. Supervised ML, particularly CNN, was the most utilized algorithm, and few of them optimized external datasets.

Current evidence has shown that upon using sufficient training datasets, various AI approaches can bring astounding performance in many tasks, such as object-identifying tasks, the main application in medical diagnosis across the reviewed literature [28]. The assistance of AI technology can make rapid detection of clinical symptoms based on the image features, like tone and rim. Computer-assisted technology is also capable of producing consistent outputs, that will lead to increasing the efficiency of healthcare service, saving vast amounts of time in clinical practice, complementing cognitive fatigue, and markedly reducing the workload of clinical practitioners. Nevertheless, the application of AI cannot be isolated from its clinical significance.

Each algorithm has its advantages and disadvantages. The conventional ones such as random forest and support vector machines might have better interpretability and be cheaper in computation cost than the deep learning-based algorithm. With that said, having expertise to decide how the features are extracted from the data is necessary. They heavily rely on such well-defined features; hence their performance is dependent on successful feature extraction. Manual feature extraction is a tedious task; therefore, many believe it is time-consuming, labor-intensive, and inflexible [29]. On the other hand, the deep learning-based algorithm can extract the feature independently. Therefore, it does not require expertise to perform the feature extraction task manually. The feature extraction and classifier are often end-to-end connected and learn together through optimization algorithms such as gradient descent. It results in a fully automated feature extraction and model training process. Therefore, it is considered the antidote to the conventional AI algorithm drawback.

In computer vision, a convolutional neural network (CNN) is a very well-known deep learning-based algorithm designed to work with grid-structured inputs, which have solid spatial dependencies in local regions of the grid such as image and video [30]. It consists of a convolution layer and pooling layer used to extract features, like edges, corners, shapes, from the input image and feed-forward it to the next layer. Each convolution layer has its parameters that can be learned during the gradient descent process; therefore, this model does not require human expertise for feature extraction since it can do the task on its own. Moreover, the shared parameters of the filter across the entire convolution make this model be equivariance to translation. In other words, if we shift an object in an image, it does not alter the representation of the data in the deeper layer of CNN. Therefore, this model often gives a promising performance.

The drawback of a deep learning-based algorithm lies in its interpretability since the model seems to learn thoroughly on its own that it is hard to explain what the model is trying to see from the data. Moreover, it is popularly known as computationally expensive that it often requires a graphical processing unit (GPU) to run the deep learning-based algorithm since it often has millions of parameters to compute. Deep learning is tough to train; even if the model managed to have 100% accuracy on the training data, it does not guarantee that the model has the same performance for unseen data. This phenomenon is called overfitting, where the model is suitable only for the training data. The simplest solution to this problem is to collect more data. With increased training data, the training accuracy will be decreased due to more diversity in the data. However, that will make the model more general, resulting in good predictions for the unseen data. The number of samples appearing for each class must be considered to avoid imbalanced class problems. An imbalanced class problem occurs when samples from a specific class occur more frequently than others. For example, if the collected data is 1000 with 700 of which are normal while 300 of which are heart failure, the model trained with this kind of imbalanced class will be biased toward the normal class, resulting in prediction attempt more frequent to the normal class than the heart failure class. This kind of model will likely have a low recall score. That is why the number of samples from each class must be considered when collecting the data.

While the advancement of AI technology might be promising, a medical evaluation by the experts still plays a vital role. The final diagnosis of the disease shall have a real-world impact to improve patients’ health; thus, AI cannot be separated from human engagement; they must work together in harmony [31]. However, AI also poses some limitations: (1) It needs high-quality datasets for training and validation; (2) There could be ethical and safety issues, e.g., using AI after obtaining the patient’s consent and determining who is liable for a misdiagnosis or incorrect treatment; and (3) it cannot determine causal relationships; thus it still need evaluation and interpretation by medical practitioners [32].

Despite having particular challenges, the future of AI in cardiology is promising in the era of precision medicine, especially in diagnosing heart failure. Heart failure has complex pathophysiology with various clinical features; thus, its diagnosis can be challenging even for cardiologists [33]. Patients with HF can have a poor prognosis and high readmission rates. The use of AI can be beneficial to rapidly detect the disease in early stages, thus improving the patient’s prognosis and saving lives. Consequently, misdiagnosis of HF can hinder the chance of improving a patient’s outcome. AI models have the potential to make better medical decisions, reduce clinical errors, and improve quality of life [34].

Even though we have covered various major databases of scientific articles, this systematic review still has the potential weakness of only including studies from the published literature and eliminating other studies, such as conference abstracts. These conference abstracts were primarily recent studies published in 2020–2022. Unfortunately, the information in these kinds of literature did not have the full study details. We might have missed some articles, especially those published in languages other than English. This systematic review highlights the need for additional research regarding the use of AI in heart failure diagnosis. Our study is the first to review the current literature on heart failure diagnosis through echocardiography and AI.

## 5. Conclusions

Cardiovascular imaging, particularly echocardiography, is an essential tool for medical practitioners, especially to detect left heart failure patients as early as possible. Studies have shown that artificial intelligence has a high potential to serve as practical auxiliary assistance for medical practitioners to differentiate normal and left heart failure patients through echocardiography. It is unlikely that artificial intelligence will completely replace cardiologists in interpreting echocardiography images, diagnostic processes, and overall clinical care. Despite limitations, AI remains a vital concept in the future of cardiology, and additional research is needed.

## Supplementary Material

Supplementary material associated with this article can be found, in the online version, at https://doi.org/10.31083/j.rcm2312402.
